# Selective Cytopheretic Device Therapy in the Context of Extracorporeal Membrane Oxygenation

**DOI:** 10.3390/medicina61091513

**Published:** 2025-08-23

**Authors:** Marton Szamosfalvi, Christopher J. Pino, H. David Humes

**Affiliations:** 1 Innovative BioTherapies, Ann Arbor, MI 48108, USAcpino@innbio.com (C.J.P.); 2 Department of Internal Medicine, University of Michigan, Ann Arbor, MI 48109, USA

**Keywords:** selective cytopheretic device, immunomodulatory device, leukocyte modulating device, extracorporeal membrane oxygenation, extracorporeal circuit, cardiopulmonary bypass, life support circuit

## Abstract

This review examines the clinical data and basic science research to evaluate the potential of the Selective Cytopheretic Device (SCD) in mitigating Extracorporeal Membrane Oxygenation (ECMO)-associated inflammation. In brief, SCD is an immunomodulatory device used within extracorporeal blood circuits along with the use of citrate anticoagulation. SCD has been shown to be a novel, first-in-its-class device (being marketed as QUELimmune by SeaStar Medical), which is capable of the autologous processing of hyper-inflamed leukocytes to reduce systemic inflammation. Strong preclinical data gathered for SCD in the context of both Cardio-Pulmonary Bypass (CPB) as well as ECMO set the stage for SCD to be used in these life support circuits. ECMO played a crucial role during the COVID-19 pandemic, during a time period when SCD therapy was being evaluated in clinical trials, generating initial clinical data in this setting. SCD has also been utilized in the setting of pediatric acute kidney injury (AKI) and multiorgan dysfunction (MOD), where ECMO can be common.

## 1. Introduction

Extracorporeal Membrane Oxygenation (ECMO) has played a crucial role in managing severe cardiac and respiratory failure, including acute respiratory distress syndrome (ARDS) during the COVID-19 pandemic [1,2]. Despite these successes, ECMO remains associated with substantial challenges impacting patient outcomes [3] and quality of life [4,5]. One challenge is the development of systemic inflammatory response syndrome (SIRS) and associated organ injury, such as acute kidney injury (AKI) [6]. This inflammatory cascade is driven by blood interaction with artificial ECMO circuit surfaces, leading to complications that can compromise patient outcomes. It has been shown that by adding a hemofilter to the ECMO circuit, it is possible to reduce the plasma concentration of interleukins [7]. Therapies attempting to utilize extracorporeal cytokine adsorption have also been used [8]. However, such interventions to reduce cytokine loads can be transient [8] and fail to address the sources of cytokines, i.e., leukocytes (LE). LE and specifically neutrophils (NEs) have been shown to be a key component of extracorporeal circuit-related inflammation [9]. This understanding was the impetus to develop therapies to mitigate LE-driven inflammatory processes in ECMO and cardiopulmonary bypass (CPB) [10]. The Selective Cytopheretic Device (SCD) is an extracorporeal, blood-contacting device filled with biocompatible membrane fibers, which has been shown to selectively adhere and sequester LE; most notably, NE and monocytes (MO) [11]. During SCD therapy, regional citrate anticoagulation (RCA) is administered [11] to create a low ionized calcium (iCa) environment for the selective sequestration of the most activated LE, as assessed by CD11b [11]. Though SCD was originally developed to reduce inflammation associated with acute kidney injury (AKI), it has strong potential use in combating other hyperinflammatory conditions [12].

This review will examine both preclinical, basic science research, as well as clinical data to evaluate the potential of the Selective Cytopheretic Device (SCD) in mitigating life support circuit-associated inflammation. Specifically, preclinical, large animal experiments will be reviewed, which established the potential efficacy of SCD in modulating the heightened immune response observed in both Cardio-Pulmonary Bypass (CPB) as well as ECMO life support circuits. SCD therapy in these contexts is also referred to as a Leukocyte Modulating Device (LMOD) in some publications. This review will report on all patients treated with SCD to date who were being supported by ECMO. This includes both pediatric and adult patient populations, which will be discussed separately. It should be understood that, similar to adults, neutrophil activation is at play in neonatal and pediatric patients on ECMO [13]; therefore, the same overall hypothesis governs all studies presented: SCD may significantly improve survival rates and overall patient outcomes by reducing excessive inflammation. ECMO circuit types will be discussed, which may influence the inflammatory response and the effectiveness of SCD therapy in this setting. Understanding these distinctions is critical in determining the optimal integration of SCD into ECMO therapy. This review aims to provide insight into how dialysis-based organ support [14] and immunomodulatory strategies [15], such as SCD, could enhance ECMO treatment, ultimately improving the clinical management of patients requiring extracorporeal life support.

## 2. Selective Cytopheretic Device (SCD) Therapy in Acute Kidney Injury (AKI)

The SCD is a first-in-its-class, autologous cell processing, immunomodulatory device, which stemmed from research and development of a renal assist device (RAD). The RAD consisted of an extracorporeal device, which included renal cell isolates from discarded donor kidneys incorporated on the inside of hollow fibers, with blood flow on the outside of hollow fibers [16]. For a comprehensive review on the transition from RAD to the discovery and early development of the SCD, please see Pino et al. [12]. In brief, like RAD, which was developed for the treatment of AKI, SCD therapy was shown to reduce mortality associated with AKI by approximately half compared to case-matched controls [17,18]. The SCD impact on the treatment of AKI and the hyperinflammation of SIRS has been well explored in other reviews [19], as well as its mechanism of action, sequestering and altering NE and MO and fundamentally shifting circulating LE to less inflamed phenotypes [20]. SCD was recently approved for use in pediatric AKI and multiorgan failure under a Humanitarian Device Exemption (HDE) by the Federal Drug Administration (FDA) and is being marketed under the trade-name QUELimmune [21]. The extension of this technology to ECMO is natural, as others have noted that AKI is common and is increasing among patients on ECMO support, along with the fact that ECMO has a high risk of mortality, and AKI is an independent predictor of mortality [22].

## 3. Preclinical Experience: SCD/LMOD in ECMO and CPB

SCD has demonstrated utility in reducing leukocyte-driven inflammation, which suggests potential utility in other indications involving hyperinflammation. Use of SCD for AKI was relegated to relatively lower blood flow rate (BFR) circuits, such as continuous kidney replacement treatment (CKRT) with maximum BFR in the 200–300 mL/min range. One fundamental hurdle to SCD integration into extracorporeal life support circuits is the higher BFR involved in these circuits, generally measured in L/min. Practical aspects of circuit integration, such as the use of shunt circuits as well as early assessments of potential immunomodulation in life support circuits, were evaluated in three different large animal studies [23,24,25]. These animal studies, while imperfect efforts to replicate the physiological responses of human patients to the combined use of SCD and life support circuits such as ECMO and CPB, do serve a crucial role that clinical studies cannot fulfill. Animal studies standardize injury conditions of the controls and the animals receiving therapy to better assess the impact of interventions. Two of the animal studies for extracorporeal life support were implemented in large animal models of CPB in bovine [24] and porcine [23] models, respectively, where, similar to ECMO, neutrophil activation also plays a key role. These studies generated preliminary data, which allowed entry into a more complex, ECMO study involving a porcine model of prolonged cardiac arrest with an alternative therapy comparator cohort, leukocyte filters [25].

## 4. Preclinical Bovine CPB Model

**Figure 1:**
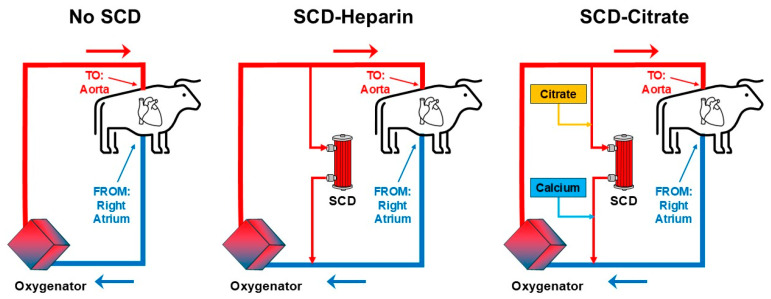
Bovine model of Cardio-Pulmonary Bypass (CPB) with the Selective Cytopheretic Device (SCD). Circuit diagrams are shown for No-SCD (negative control), SCD-Heparin (SCD-H), and SCD-Citrate (SCD-C) groups. Parallel circuits incorporating SCD utilized a regional flow rate of 200 mL/min.

In the bovine model of CPB, meant to explore the hypothesis that SCD could reduce SIRS-like inflammation after CPB under ex vivo experimental conditions, the choice of an appropriate control group was a key factor. SCD therapy consists of two important aspects: biomimetic fibers, which mimic capillary beds for the sequestration of activated leukocytes, and pharmacologic intervention in the form of regional citrate anticoagulation, which reduces ionized calcium. To decouple these two aspects, a control group was chosen, which utilized fiber-filled devices, but treatment was administered using systemic heparin anticoagulation (SCD-H), rather than regional citrate anticoagulation, referred to as SCD-citrate (SCD-C) in this study [24]. The No-SCD group, which was a standard CPB circuit without any additional devices, was used as a negative control group. The circuit diagrams for each of these groups are shown in Figure 1.

The study’s central thesis was that through the contact and shear stress of the patient’s blood coming into contact with the circuit of the CPB, there is a SIRS response that manifests with LE count elevation and activation, particularly NE [24]. The increase in activated neutrophil levels can play a role in organ injury, particularly acute kidney injury (AKI) and multi-organ dysfunction syndrome (MODS), which can lead to increased complications post-CPB surgery. Analysis by Salis et al. found postoperative renal complications in 7.8% of the CPB patients studied and postoperative multiorgan failure in 1.6% [26]. A meta-analysis published in 2015 looked at 242,388 patients across 47 studies that had undergone CPB; the pooled rate of CPB-associated AKI was 18.2% and 2.1% of the patients ended up requiring renal replacement therapy [27]. CPB-related AKI was associated with an elevated early mortality risk ratio of 4.0 [27].

In the bovine model of CPB, SCD therapy (SCD-C), control fiber devices without the low iCa afforded by RCA (SCD-H), and the negative control (No-SCD) groups were fundamentally different. The No-SCD negative control group showed a continuous rise in systemic WBC and neutrophil counts throughout the experiment that began ~60 min after CPB initiation [24]. The SCD-H and SCD-C groups stayed very close to each other regarding systemic WBC and neutrophil levels until ~150 min [24]. Interestingly, after this time, there was a considerable increase in WBC and NE for the SCD-H relative to the SCD-C group, which was associated with an influx of immature NE. SCD-C and SCD-H devices were routinely removed at T = 225 min, with a final blood sample taken 15 min after removal to evaluate post-therapy dynamics. It was shown that for SCD-C, post-therapy NE remained low, demonstrating a durable impact on NE reduction, whereas when SCD-H was removed, NE only further increased [24]. In this same report, some in vitro testing with abattoir blood was performed to compare SCD and the Pall LGB leukoreduction filter. It was demonstrated that SCD treatment generated lower amounts of myeloperoxidase, a generalized inflammation marker in blood, compared to leukoreduction filters [24]. Also, more LE were unaccounted for in pre- and post-therapy measurements, as well as adherent cell quantification in the leukoreduction filter group, which is potentially suggestive of loss of cells from circuits by destruction. This was suggestive that the way in which SCD reduced LE was different from that in leukoreduction filters, and that SCD was likely better at reducing inflammation in blood.

## 5. Preclinical Porcine CPB Model

**Figure 2:**
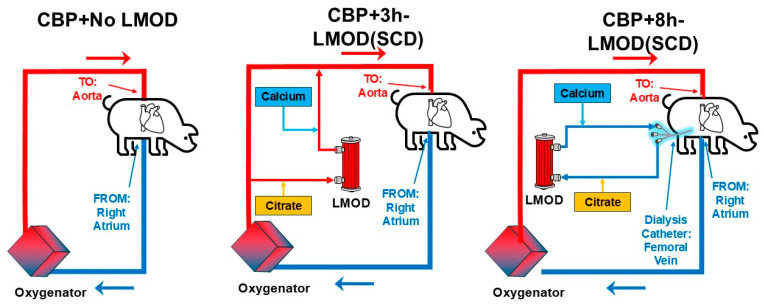
Circuit diagrams in the porcine model of Cardio-Pulmonary Bypass (CPB) to evaluate the SCD, which was renamed a Leukocyte Modulating Device (LMOD) when used in life support circuits. Circuits studied included No-LMOD, negative control (left), 3 h-LMOD therapy within an integrated CPB circuit (middle), and 8 h-LMOD therapy via a CKRT circuit separate from the CPB circuit.

Building upon an understanding of the necessity of regional citrate coagulation with SCD from the previous animal study, there was a desire to understand the ideal placement and duration of the SCD intervention in conjunction with CPB. A practical method of SCD use during and possibly after CPB needs to be elucidated to maximize the reduction in the SIRS development observed in CPB patients. With this new approach to using the SCD in life support circuits, our group renamed the device and associated therapy as a Leukocyte Modulatory Device (LMOD) when used in this context. LMOD was evaluated in two experimental groups. In the CPB-Access group, the LMOD was incorporated directly into the CPB circuit, allowing only for therapy during 3 h of CPB treatment [23]. In the CKRT-Access group, blood access was obtained via a dialysis catheter in a central vein, separate from the primary CPB circuit, establishing a CKRT-RCA circuit. This allowed the SCD therapy to run for the 3 h of CPB, as well as an additional 5 h after the CPB circuit had been discontinued [23]. In addition to the two experimental groups, a CPB-Only control group was treated with just the standard CPB circuit and no SCD incorporation. Circuit diagrams for each are shown in Figure 2.

The CKRT-Access group treated with the LMOD(SCD) during CPB and in the 5 h post-CPB had significantly lower levels of LE, including total NE, and immature NE across all times post-CPB [23] compared to the other groups. These data suggest there may be a substantial benefit from delivering continuous SCD therapy from a separate circuit to quell the inflammation caused during CPB and in the period after CPB discontinuation. In practice, clinicians may find it inconvenient to have to cannulate a central vein and establish a separate blood access for SCD + CKRT (with RCA) circuit apart from the CPB circuit. However, this approach allows for vastly improved flexibility in terms of SCD treatment options and duration, even allowing for options to pre-treat LE prior to CPB initiation, full duration treatments while establishing and weaning CPB, and treatment after CPB. Optimal LMOD therapy in terms of optimized start time and duration to improve outcomes in CPB still needs to be explored more fully.

## 6. Preclinical Porcine Model of Extracorporeal Cardiopulmonary Resuscitation (EPR) Supported with ECMO

**Figure 3:**
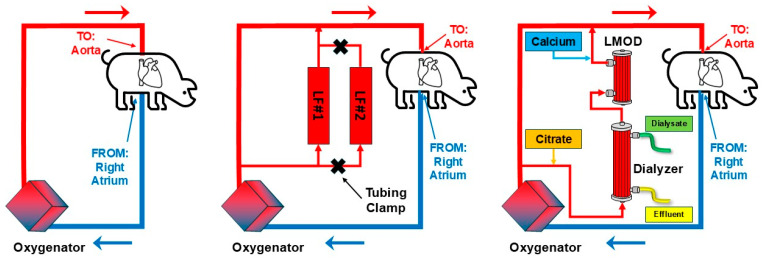
Circuit diagrams for therapies examined in a porcine model of ECPR supported by ECMO. Circuits studied included standard ECMO circuit without additional devices (left), leukoreduction filters integrated within an ECMO circuit (middle), and LMOD/SCD in series after a dialyzer, integrated within an ECMO circuit (right).

Though CPB and ECMO are both forms of life support with some commonalities, a key difference is that ECMO is typically used for days to weeks, whereas CPB, which is generally only used for a few hours [28]. Previous animal studies examining the SCD/LMOD and extracorporeal circulatory support were constrained to shorter CPB sessions. In this study, our group collaborated with the ECMO lab at the University of Michigan. Swine underwent 8 min of untreated ventricular fibrillation cardiac arrest, followed by 30 min of mechanical cardiopulmonary resuscitation (CPR) and a subsequent 8-h period of Extracorporeal Cardiopulmonary Resuscitation (ECPR) from an ECMO circuit [25]. There were three cohorts studied, including treatment with a standard ECMO circuit, and those that also received either two leukoreduction filters (Pall LS) or those that received a dialyzer and LMOD/SCD. Circuit diagrams for these treatment groups are shown in Figure 3.

Despite its widespread implementation, one of the major obstacles to successful organ recovery in ECPR supported by ECMO remains microvascular no-reflow [2]. This condition, where circulation fails to return to the microvasculature after reperfusion, can prevent organs from regaining full function even after restoring blood flow. One suspected mechanism behind this no-reflow state involves the role of leukocytes, particularly the formation of neutrophil extracellular traps (NETs) [25]. NETosis, the process by which neutrophils release DNA and enzymes to trap pathogens, has also been implicated in tissue damage and impaired reperfusion. Based on this understanding, researchers set out to examine whether targeting leukocytes during ECPR could help reduce NETosis and promote better heart and brain function recovery [25]. Blood samples were collected to measure biomarkers associated with NETosis, including double-stranded DNA and citrullinated histone. Tissue samples from the heart and brain were also analyzed for evidence of microvascular NET formation through immunofluorescence staining [25].

In all groups, there was an increase in NETosis markers following cardiac arrest and reperfusion, and both the heart and brain showed signs of NET accumulation in the microvasculature. Despite these findings, neither the LF nor the SCD/LMOD interventions produced a noticeable reduction in NETosis [25]. Furthermore, when recovery outcomes were assessed, animals in the treatment groups did not show significant improvement in cardiac or neurologic function compared to those who received standard ECPR care. Measurements such as the cardiac resuscitability score and cortical responses in somatosensory evoked potentials suggested no meaningful therapeutic benefit from either device [25]. While the underlying hypothesis that reducing NETosis improves tissue reperfusion and function still remains plausible, the specific interventions tested in this study did not achieve the desired effect.

Several limitations may have influenced the study’s conclusions. When this study was initiated, final results from the porcine model of CPB had not yet been collated, and it was not yet known that the administration of LMOD/SCD therapy from a separate circuit might be more effective. This form of LMOD therapy may have been more amenable in the ECPR model, but then it would not have been an analogous circuit to the one used to assess leukoreduction filters. Other limitations of the study included a relatively short duration of the post-resuscitation observation period, making it challenging to assess longer-term impacts of the interventions [25]. In addition, while the study focused on NETosis as a proxy for microvascular dysfunction, it did not directly measure the extent of no-reflow in the tissue, leaving some uncertainty about the actual vascular dynamics. It is also possible that the degree of NET formation in this model was not severe enough to allow the effects of the treatments to be detected [25]. Lastly, although swine models are valuable for simulating human physiology, they do not fully replicate the complex clinical conditions under which ECPR is typically used in human patients [25]. In conclusion, while the concept of targeting leukocyte activity to improve organ recovery after cardiac arrest remains compelling, the use of LF and SCD/LMOD in this study did not reduce NETosis nor enhance recovery of heart or brain function. These findings underscore the need for continued investigation into more effective strategies to mitigate reperfusion injury and improve outcomes in ECPR, as well as highlight the challenge of conducting complex, large animal models. Lastly, it illustrates difficulties involved with trying to evaluate emerging technologies, such as LMOD, which have not yet been optimized for varied applications.

## 7. Pediatric Clinical Data

The very first use of SCD on a patient undergoing ECMO was during the SCD-PED-01 clinical trial (NCT02820350), evaluating the safety and efficacy of SCD in pediatric AKI, where patients were <22 years old and had a dry weight of >20 kg. A pediatric patient undergoing ECMO was also treated with SCD during the SCD-PED-02 clinical trial (NCT04869787). These were both treatment arm only studies, which collectively enrolled 22 patients with AKI requiring CKRT, many of whom had SIRS or sepsis [29]. Previously, ECMO patients were excluded from SCD studies due to concerns over hemodynamic instability and the risk of complex interactions between multiple extracorporeal circuits having the ability to influence study outcomes and possibly cause adverse events in patients. However, in light of ECMO’s expanding role in pediatric critical care, particularly for refractory septic shock and multi-organ dysfunction, ECMO patients were included. This regulatory flexibility acknowledged the real-world overlap between CKRT and ECMO support in the pediatric intensive care unit (PICU). It opened the door to exploring novel immunomodulatory therapies in this vulnerable cohort.

Of the 22 patients enrolled across both trials, 4 (18.2%) received concurrent ECMO and SCD + CKRT-RCA therapy—3 in SCD-PED-01 and 1 in SCD-PED-02 [30]. All four of these ECMO + SCD patients survived to ICU-discharge, suggesting a potentially favorable risk–benefit profile of SCD use in this setting [29]. This is particularly noteworthy given the high mortality traditionally associated with pediatric patients requiring both CKRT and ECMO, where mortality can exceed 50% depending on the underlying etiology and timing of organ support initiation. In conjunction, among the five total ICU non-survivors in the combined studies, four had received ECMO at some point during their PICU stay—three during concurrent CKRT-SCD therapy and one after SCD therapy had ended [29]. In this context, it is crucial to understand that the disproportionate presence of ECMO therapy amongst ICU non-survivors suggests a higher acuity level among non-survivors while receiving SCD treatment. The higher acuity level for the simultaneous ECMO and SCD may have prevented meaningful, interpretable results that could allude to the immunomodulatory effects of SCD in the most critically ill PICU patients.

Crucially, the main result of significance generated from the study is that no SCD-related adverse events were reported among the ECMO-treated patients, underscoring the safety of incorporating SCD into the CKRT circuit used simultaneously with ECMO. This aligns with prior adult studies suggesting that SCD use can be safely integrated into complex extracorporeal circuits, modulating excessive inflammation by selectively deactivating primed neutrophils and monocytes without compromising immune surveillance. While limited in size, these studies offer promising evidence supporting the feasibility and safety of SCD therapy in pediatric patients requiring ECMO. Since the approval of SCD (QUELimmune) through the FDA’s HDE mechanism, a pediatric surveillance registry has been established (NCT06517810) for all patients < 22 years and > 10 kg treated.

## 8. Clinical: Adult Patient Data Gathered to Date

The COVID-19 pandemic presented a unique opportunity to evaluate the therapeutic potential of the SCD in critically ill patients with Acute Respiratory Distress Syndrome (ARDS), as an initial two patients were successfully treated via Emergency Use Authorization (EUA) [31]. This led the way toward an Investigational Device Exemption (IDE) approval to study SCD in a clinical trial on COVID-19 patients (NCT04395911). Due to the prevalence of ECMO in this patient population, for the first time, an adult clinical study was designed to allow the combined use of SCD and ECMO in intensive care unit (ICU) patients. Before this, ECMO patients had been systematically excluded from SCD clinical trials due to circuit complexity and patient stability concerns.

This multicenter observational study enrolled 38 adult COVID-19 patients with severe respiratory failure—22 in the SCD-treated group and 16 in a contemporaneous control group—at the University of Michigan and the University of Kentucky [32]. Nearly all patients in the SCD-treated group (with the exception of one) developed an AKI requiring CKRT. The SCD was integrated into the CKRT circuit for up to 10 days in the treated cohort to mitigate the systemic inflammatory response syndrome (SIRS) and cytokine storm associated with acute respiratory distress syndrome (ARDS) in severe COVID-19 cases.

Among the total study patient cohort of 38, 13 required ECMO support during their ICU stay. Of this group, 9 ECMO patients were treated with SCD, while 4 were in the control group and did not receive SCD (11). The survival outcomes were as follows: none of the ECMO patients in the control group survived, whereas four out of the nine SCD-treated ECMO patients (44%) survived to ICU discharge [32]. This differential suggests a potential survival benefit associated with SCD therapy when used in combination with ECMO and CKRT in the context of COVID–19-related multi-organ dysfunction syndrome.

These findings provided the first clinical insight into the integration of SCD therapy within the CKRT circuit, along with an ECMO circuit, and highlight its potential role in attenuating overwhelming immune responses. By modulating activated leukocytes, the SCD may have helped to interrupt the cascade of cytokine-driven injury that often complicates severe viral infections, such as in COVID-19. While the sample size remains limited and further randomized studies are needed to validate efficacy, the absence of SCD-related adverse events and the observed potential survival advantage in the SCD-treated ECMO group support the continued exploration of this therapy in critically ill patients experiencing inflammatory organ dysfunction.

To date, over 250 patients, including both pediatric and adult patients, have been treated with the SCD, along with approximately half of an additional 100 patients enrolled in an ongoing clinical trial [33]. So far, no severe adverse events attributable to the SCD have been reported [21]. One safety concern that existed (due to a lack of clinical trial data) regarding two highly relevant clinical parameters was the application of the SCD in patients with low platelet counts (thrombocytopenia) and low circulating neutrophils (neutropenia). A recent case study in a pediatric patient suggests that SCD may be able to successfully treat such patients [34] without safety issues.

According to a recent meta-analysis, the pooled prevalence of thrombocytopenia in ECMO patients was 21% [35]. The meta-analysis looked at studies dated from 1975 to 2019. A more recent study conducted between 2016 and 2019 showed that in a clinical cohort of 67 ECMO patients, 26 (38.8%) were developing severe thrombocytopenia. Patients developing severe thrombocytopenia had a 3.65-fold increased risk of a thrombotic event occurring and a significant decrease in in-hospital survival (odds ratio 0.288) [36]. Thrombocytopenia, in and of itself, is widely used as one of the prognostic indicators for SOFA scoring in multi-organ failure patients.

Within the context of ECMO patients, there can also be an intriguing combination of both thrombocytopenia and neutropenia that can make managing systemic inflammation a complex clinical task. For context, ECMO is commonly used as a bridge to transplant for lung failure. An analysis of adult patients receiving ECMO as a bridge to transplant (BTT) at Columbia University Medical Center (a high-volume lung transplant and ECMO center) between 2009 and 2019 showed that 13.8% of all lung transplant patients were bridged to transplant with ECMO [37]. Since the advent of COVID-19 and the broader acceptance of the utility of ECMO, the share of lung transplant patients using ECMO as a BTT is only expected to increase. Neutropenia is a common adverse condition that can occur in lung transplant patients as a result of the immunosuppressants given during the pre-operative and post-transplant period. Some studies have shown the incidence of neutropenia to be as high as 51.8% amongst lung transplant patients post-transplant, with 12.54% developing severe neutropenia [38].

## 9. Discussion

### 9.1. Issues in Clinical Trial Design

To date, there has been no specific clinical trial designed to interrogate the use of SCD during ECMO within various subpopulations; there have been several case study experiences collected through the conduct of other indication-specific trials. All use of SCD in ECMO patients has been due to AKI or ARDS, and ARDS patients were limited to adults with COVID-19. Within pediatric trials, such as SCD-PED-01 and SCD-PED-02, with an indication that qualifies for orphan disease designation (as is the case for pediatric AKI), such trials face limited enrollment. Trial design options can be limited to intervention-arm only studies with case-matching rather than controlled trials with contemporaneous controls [39]. There are several ethical considerations in trials like these, where there can be a significant probable benefit of experimental therapy, and alternative approved treatments are not available or severely limited. In such trials, there can be significant variance in the prognosis and survivability of patients and perfect case-matching can be difficult; contemporaneous control trials can be impossible to conduct. Despite the gold standard of controlled, randomized, multicenter, blinded trials, fulfilling all of these aspects is not always possible. Blinding can be difficult with extracorporeal therapies, which include circuit elements that need to be visually monitored by perfusionists. Despite gains in the availability of life-saving ECMO therapy [40,41], its use is not yet ubiquitous due to the complexities involved, which can limit the number of institutions able to participate, making it more difficult to conduct multicenter trials. Within trials related to ECMO, as Del Marmol et al. have noted, “even well conducted trials face limitations, including heterogeneous patient populations and challenges in endpoint selection, making definitive conclusions difficult” [34]. With such heterogeneous clinical conditions and varied risk factors [42], assessing SCD efficacy in the context of ECMO would require a very careful study design to conduct valid controlled studies.

### 9.2. SCD/LMOD Addresses Several Mechanisms of Action

ECMO and CPB both provoke a biomaterial-induced inflammatory response due to blood exposure to extracorporeal circulation. However, key differences between these life support techniques must be considered when comparing their inflammatory effects. The inflammatory cascade triggered by ECMO resembles SIRS and is driven by complex interactions between humoral and cellular mechanisms. Major contributors include the contact system, coagulation pathways, complement system, endothelial cells, leukocytes, platelets, and cytokines. Although these components function interdependently, their roles in ECMO-induced inflammation warrant separate analysis [24]. For the purpose of assessing the role of adding SCD to various extracorporeal treatments (ECMO or CPB), a central component can be the presence of tissue injury. Ischemia–reperfusion injury (IRI) significantly influences this inflammatory response, further modulating immune system activity.

The underlying pathology necessitating ECMO support, as well as prior cardiac surgery, can also impact immune function, though to a lesser extent. Despite extensive research, the precise mechanisms underlying ECMO-induced inflammation remain incompletely understood. The concept of a two-hit model in immune activation offers valuable insight into refining therapeutic strategies for optimizing patient outcomes. CPB commonly lasts for 1 to 2 h, while the median duration of ECMO is four days [24]. Additionally, CPB necessitates a bypass of the heart and lungs, resulting in ischemia in these organs. Following the conclusion of CPB, reperfusion occurs, initiating an ischemia–reperfusion process that will be explained later. Given the multi-organ ischemia until ECMO initiation, a similar process can occur, albeit to a lesser extent, during ECMO [24]. In these scenarios, the deactivation of circulating neutrophils via the SCD may significantly reduce tissue injury resulting from ischemia–reperfusion.

The SCD, which deactivates circulating neutrophils, may offer a promising strategy to modulate inflammatory responses by reducing neutrophil extracellular trap (NET) formation through the inhibition of NETosis. By limiting NET production, the SCD could decrease the harmful effects of excessive neutrophil activation, which is often observed in inflammatory conditions [25]. Moreover, independent of its impact on NETosis, the SCD has been shown to reduce TNF-α levels in treated patients, thereby alleviating excessive immune activation and systemic inflammation. The effectiveness of the SCD in modulating immune responses, particularly in the context of TNF-α, is a key area of interest. TNF-α is a crucial immune regulatory cytokine produced by various cells in response to inflammatory stimuli. It plays a vital role in neutrophil activation, endothelial adhesion molecule expression, macrophage phagocytosis, prostaglandin production, and thrombin formation. During ECMO, TNF-α is initially released from pre-formed stores within mast cells. This elevated TNF-α contributes to immune dysregulation, potentially worsening patient outcomes. The suppression of TNF-α levels by the SCD could be particularly beneficial in ECMO patients, where heightened TNF-α levels are linked to disease progression and poor prognosis [43]. If SCD effectively mitigates TNF-α-driven inflammatory damage, it could improve survival rates by preventing the cascade of immune dysfunction associated with ECMO-related complications. Further studies are needed to determine whether SCD-mediated neutrophil deactivation and NET suppression translate into clinically significant benefits for ECMO patients.

Immunomodulation via SCD presents a promising therapeutic approach for mitigating excessive inflammation in conditions like sepsis and during CPB or ECMO use. By selectively deactivating neutrophils that produce pro-inflammatory cytokines like TNF-α, the SCD may help restore immune balance, reduce systemic inflammation, and prevent excessive neutrophil activation. This could enhance microvascular function, improve tissue perfusion, and potentially decrease the risk of multi-organ failure. Elevated TNF-α levels have been correlated with poor survival outcomes in neonates undergoing ECMO, suggesting that high TNF-α may serve as a biomarker of disease severity [43]. Additionally, by promoting the clearance of circulating NETs and cell-free DNA, SCD therapy may help reduce the inflammatory burden associated with NET-driven pathology, improving clinical outcomes in critically ill patients [25].

### 9.3. LMOD/SCD Circuit Considerations

**Figure 4:**
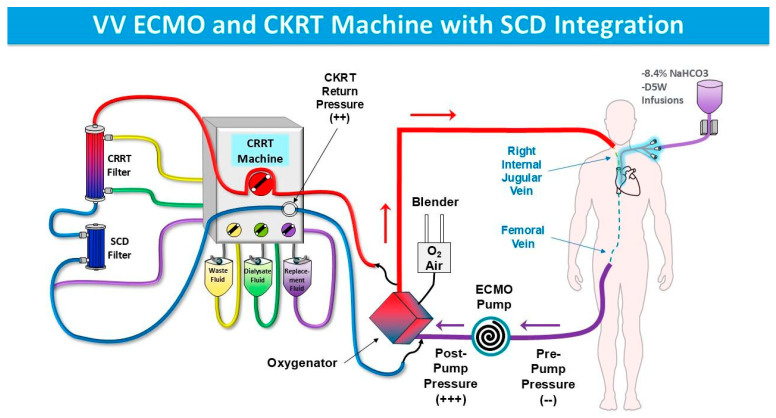

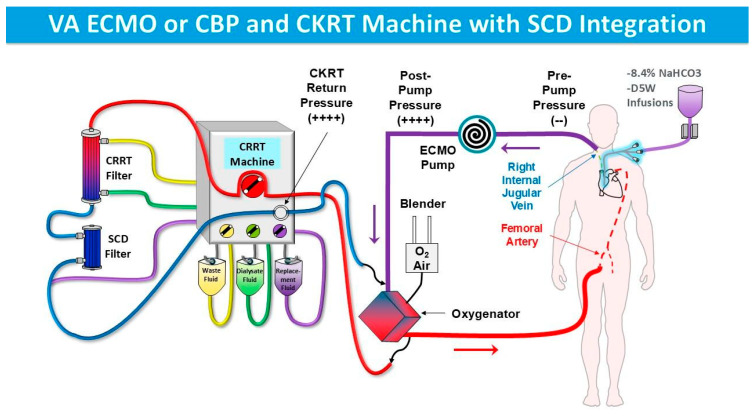
SCD integration in the context of veno-venous (VV) ECMO (top) and veno-arterial (VA ECMO) (bottom).

There is strong evidence that LMOD modulates the inflammatory response in ECMO patients by deactivating circulating neutrophils, thereby reducing excessive inflammation and preventing tissue damage. Bradykinin, a key inflammatory mediator, is crucial in neutrophil recruitment, activation, and chemotaxis. By increasing vascular permeability and promoting the extravasation of neutrophils into surrounding tissues, bradykinin amplifies the inflammatory response [44]. In ECMO patients, particularly those on veno-arterial (VA)-ECMO, bradykinin levels are expected to be higher due to the direct re-infusion of blood returning from the ECMO unit into the systemic circulation without passing through the lungs, where bradykinin is degraded by the angiotensin-converting enzyme (ACE) [37]. This elevated systemic bradykinin environment may lead to increased neutrophil activation and adhesion to endothelium, contributing to ECMO-associated SIRS [44]. Given this interplay, the effectiveness of the SCD in deactivating neutrophils may be influenced by the extent to which bradykinin drives their activation and migration. In the presence of high bradykinin levels, neutrophils may be more prone to activation, potentially counteracting the intended immunomodulatory effects of SCD. While SCD functions by deactivating neutrophils in the circulation, bradykinin-driven endothelial activation may lead to increased sequestration of neutrophils within tissues, reducing the pool of circulating neutrophils available for SCD-mediated modulation. This effect would be expected to be more pronounced in VA-ECMO, where bradykinin’s systemic vasodilatory and permeability-enhancing effects are greater than in veno-venous (VV)-ECMO [44]. However, if the SCD successfully reduces circulating neutrophil activation early in the inflammatory cascade, it may indirectly attenuate bradykinin-driven tissue infiltration by limiting the overall inflammatory response. Therefore, understanding the relationship between bradykinin levels, neutrophil behavior, and SCD efficacy may be critical for optimizing its therapeutic application in ECMO patients. Figure 4 demonstrates how SCD may be able to be integrated into VV and VA-ECMO circuits, respectively.

### 9.4. Future Directions: Use of ECMO and SCD to Improve Transplantation

ECMO and SCD may play various and important future roles in organ transplantation, in pre-transplant, peri-transplant, and post-transplant phases [45]. The pre-transplant phase entails improving patient stability for better outcomes after transplantation. The pre-transplant phase entails improving donor organ viability and reducing the number of inflammatory leukocytes within donated tissues. The post-transplant phase entails reducing inflammation after surgery, reducing graft rejection, and improving organ survival. One of the key benefits of ECMO is its ability to buy time for patients to receive a heart- and/or lung transplant that can result in survival with a good quality of life for years afterward [46]. The SCD may affect SIRS in a way that leads to a lower incidence of severe AKI and may even enhance recovery from AKI, leading to overall better outcomes. A collection of case reports [45] relevant to the impact of SCD on successful organ transplant included a patient with hemophagocytic lymphohistiocytosis (HLH) who survived to bone marrow transplantation, a patient with hepatorenal syndrome (HRS) who survived to liver transplant, and a chronic heart failure patient who was bridged to left ventricular assist device (LVAD) implantation [47]. The potential benefits of applying the SCD in the treatment of ECMO patients are summarized based on the SCD’s ability to ameliorate the heightened inflammatory response before and after the patient receives the transplant. Changes in inflammatory markers due to combining SCD support with ECMO suggest that the SCD can mitigate the overactive innate immune response present prior to transplantation and may also be observed after transplantation.

## 10. Conclusions

Further research is essential to understand the full scope of SCD/LMOD-mediated immunomodulation and its long-term benefits in life support circuits such as CPB and ECMO. Data generated to date suggest that SCD/LMOD can be safely integrated into life support circuits, and there is a preliminary signal that SCD/LMOD may be able to improve patient outcomes; however, much more work remains to be done to confirm the efficacy of this approach.

## Abbreviations

ACE	angiotensin converting enzyme
AKI	acute kidney injury
ARDS	acute respiratory dysfunction syndrome
BFR	blood flow rate
BTT	bridge to transplant
CKRT	continuous kidney replacement therapy
CPB	cardiopulmonary bypass
CPR	cardiopulmonary resuscitation
CVVH	continuous veno-venous hemofiltration
ECMO	Extracorporeal Membrane Oxygenation
ECPR	extracorporeal cardiopulmonary resuscitation
ESRD	end stage renal disease
EUA	emergency use authorization
FDA	Federal Drug Administration
HDE	humanitarian use device exemption
HLH	hemophagocytic lymphohistiocytosis
HRS	hepatorenal syndrome
iCa	ionized calcium
ICU	intensive care unit
IDE	investigational device exemption
IRI	ischemia reperfusion injury
LE	leukocyte
LF	leukofilter
LMOD	Leukocyte Modulating Device
LVAD	left ventricular assist device
MO	monocyte
MOD	multiple organ dysfunction
MODS	multiple organ dysfunction syndrome
MOF	multiple organ failure
NET	neutrophil extracellular trap
NICU	neonatal intensive care unit
NE	neutrophil
PICU	pediatric neonatal intensive care unit
RAD	Renal Assist Device
RCA	regional citrate anticoagulation
SCD	Selective Cytopheretic Device
SIRS	systemic inflammatory response syndrome
TNFα	tumor necrosis factor α
VA ECMO	venous–arterial ECMO
VV ECMO	veno-venous ECMO
